# Social disparities in food preparation behaviours: a DEDIPAC study

**DOI:** 10.1186/s12937-017-0281-2

**Published:** 2017-09-20

**Authors:** Caroline Méjean, Wendy Si Hassen, Séverine Gojard, Pauline Ducrot, Aurélie Lampuré, Hans Brug, Nanna Lien, Mary Nicolaou, Michelle Holdsworth, Laura Terragni, Serge Hercberg, Katia Castetbon

**Affiliations:** 1 Université Paris 13, Sorbonne Paris Cité, Equipe de Recherche en Epidémiologie Nutritionnelle, Centre de Recherche en Epidémiologies et Biostatistiques, Inserm (U1153), Inra (U1125), Cnam, F-93017 Bobigny, France; 2 INRA, UMR 1110 MOISA, F-34000 Montpellier, France; 3 INRA, UR1303 ALISS, 65 Boulevard de Brandebourg, F-94205, Ivry sur Seine Cedex, France; 4 Department of Epidemiology & Biostatistics, EMGO Institute for Health and Care Research, VU University Medical Center, van der Boechorststraat 7, 1081 BT Amsterdam, The Netherlands; 5 Department of Nutrition, Faculty of Medicine, University of Oslo, P.O. Box 1046 Blindern, -0316 Oslo, NO Norway; 6 Department of Public Health, Academic Medical Centre, University of Amsterdam, Amsterdam, The Netherlands; 7 School of Health and Related Research (ScHARR), Public Health section, The University of Sheffield, Sheffield, 30 Regent Street, Sheffield, S1 4DA UK; 8 Department of Nursing and Health Promotion, Faculty of Health Sciences, Oslo and Akershus University College of Applied Sciences, Oslo, Norway; 9 Université Libre de Bruxelles (ULB), Ecole de Santé Publique, Route de Lennik 808, B-1070, CP 598 Bruxelles, Belgium

**Keywords:** Food preparation, Cooking practices, Socioeconomic, Cooking skills

## Abstract

**Background:**

The specific role of major socio-economic indicators in influencing food preparation behaviours could reveal distinct socio-economic patterns, thus enabling mechanisms to be understood that contribute to social inequalities in health. This study investigated whether there was an independent association of each socio-economic indicator (education, occupation, income) with food preparation behaviours.

**Methods:**

A total of 62,373 adults participating in the web-based NutriNet-Santé cohort study were included in our cross-sectional analyses. Cooking skills, preparation from scratch and kitchen equipment were assessed using a 0–10-point score; frequency of meal preparation, enjoyment of cooking and willingness to cook better/more frequently were categorical variables. Independent associations between socio-economic factors (education, income and occupation) and food preparation behaviours were assessed using analysis of covariance and logistic regression models stratified by sex. The models simultaneously included the three socio-economic indicators, adjusting for age, household composition and whether or not they were the main cook in the household.

**Results:**

Participants with the lowest education, the lowest income group and female manual and office workers spent more time preparing food daily than participants with the highest education, those with the highest income and managerial staff (*P* < 0.0001). The lowest educated individuals were more likely to be non-cooks than those with the highest education level (Women: OR = 3.36 (1.69;6.69); Men: OR = 1.83 (1.07;3.16)) while female manual and office workers and the never-employed were less likely to be non-cooks (OR = 0.52 (0.28;0.97); OR = 0.30 (0.11;0.77)). Female manual and office workers had lower scores of preparation from scratch and were less likely to want to cook more frequently than managerial staff (*P* < 0.001 and *P* < 0.001). Women belonging to the lowest income group had a lower score of kitchen equipment (*P* < 0.0001) and were less likely to enjoy cooking meal daily (OR = 0.68 (0.45;0.86)) than those with the highest income.

**Conclusion:**

Lowest socio-economic groups, particularly women, spend more time preparing food than high socioeconomic groups. However, female manual and office workers used less raw or fresh ingredients to prepare meals than managerial staff. In the unfavourable context in France with reduced time spent preparing meals over last decades, our findings showed socioeconomic disparities in food preparation behaviours in women, whereas few differences were observed in men.

**Electronic supplementary material:**

The online version of this article (10.1186/s12937-017-0281-2) contains supplementary material, which is available to authorized users.

## Background

A shift in food preparation and cooking practices has emerged in recent decades in industrialized countries, including France [1–5]. This change could have an impact on dietary quality and therefore health, since previous studies have shown that some dimensions of food preparation behaviours are associated with poorer adherence to nutritional guidelines, higher intakes of fat and lower intakes of fruits and vegetables, folate, and vitamin A [6–14]. Food preparation behaviours are complex to define and the research literature reports on a range of indicators to assess food preparation [5, 15]. Most studies assessed food preparation behaviours by measuring time spent on food preparation [1, 3, 5, 7, 11, 14–20] and cooking skills and knowledge [5–7, 9, 13, 18, 21–24]. Some authors were interested in enjoyment of cooking [9, 13, 18, 24, 25], others studied use of raw or fresh ingredients requiring no or minimal processing [7, 13, 25–27], or the complexity of food preparation [10, 13]. However, most studies used only one dimension to capture food preparation behaviours.

Unlike socioeconomic disparities in foods and nutrient intake, socioeconomic differences for food preparation behaviours have been explored less, although they might contribute to health inequalities within the population by influencing diet quality. Available studies of food preparation have shown inconsistent evidence of socioeconomic differences in time spent on food preparation [11, 16, 19, 20, 28], cooking skills [12, 21, 23], cooking interest [8, 25], preparation from scratch and complexity level for preparation [25, 26]. However, few of these studies used several socio-economic (SEP) indicators together [12, 16, 19, 20, 28], although the relationship between each of the three major SEP indicators (education, occupation and income) with food preparation behaviours may be independent of the two other SEP factors [29, 30]. For instance in women, when adjusted for education and employment status, income was found to be unrelated to time spent preparing food [20], while a majority of studies taking only one SEP indicator into account have found that individuals with low income spend more time cooking than those with a higher income [3, 19, 31]. Indeed, education, income and occupation are not interchangeable and can even have additive or synergistic effects on dietary behaviours, although they are correlated [29, 30, 32]. The fact that correlations between SEP indicators are generally modest suggests some shared association but also that they are conceptually distinct, and their influence is transmitted by different social processes [32]. In fact, education is linked to dietary behaviours through knowledge and attitudes, while income reflects financial means and occupation can reflect one’s social network [33]. Regarding food preparation, higher income may be positively associated with more kitchen equipment and greater use of takeaways, leading to lower time frequency and a lack of association with enjoying cooking and cooking skills. Occupational level is a measure of social capital and prestige [33]. Individuals with high social capital can be expected to have lifestyle values emphasizing social interaction [34]. The information potential of one’s network may provide advice and socialize one’s interest in cooking. Alternatively, greater social capital may involve a higher likelihood of cooking for family and friends out of routine rather than for pleasure, which influences the time for meal preparation. Besides, knowledge attained through education may contribute towards individuals being skilled to prepare healthy meals from scratch, enhancing their capacity to generate healthy dietary behaviours [35, 36].

As different SEP indicators might affect food preparation behaviours differently, to study the respective role of each indicator is useful to identify population subgroups with poor food preparation behaviours, which may help public health policymakers to target populations more efficiently. The aim of the study was therefore to assess which SEP indicators are independently associated with various dimensions of food preparation behaviours in French adults by including simultaneously in the models education, occupation and income.

## Methods

### Study population

This cross-sectional analysis focused on participants of the NutriNet-Santé Study, a large web-based prospective observational cohort launched in France in May 2009 among volunteers from the general population of internet-using adults (> = 18 y). The cohort was designed to investigate the relationship between nutrition and health, as well as determinants of dietary behaviour and nutritional status. The design, methods and rationale have been described in detail elsewhere [37]. Briefly, eligible participants were recruited by a variety of means. Initially a vast multimedia campaign (television, radio, national and regional newspapers, posters, and internet) called for volunteers and provided details on the study’s specific website (http://www.etude-nutrinet-sante.fr). Then, multimedia campaigns were repeated every six months. Further information is maintained on a large number of websites (national institutions, city councils, private firms, web organizations). A billboard advertising campaign is regularly updated via professional channels (e.g. doctors, pharmacists, dentists, business partners, municipalities). In order to be included in the cohort, participants had to complete a set of questionnaires assessing dietary intake, physical activity, anthropometry, smoking and socio-economic conditions, along with health status at baseline and each subsequent year. Additionally, each month participants were invited to complete complementary questionnaires related to the food behaviours, nutritional and health status. Food preparation behaviours were assessed after 22 months of inclusion in the NutriNet-Santé study using an original web-based questionnaire. Socio-economic position and demographic data were collected at 24 months after inclusion, which is close to the administration of the food preparation questionnaire.

This study was conducted according to guidelines laid down in the Declaration of Helsinki, and all procedures were approved by the Institutional Review Board of the French Institute for Health and Medical Research (IRB Inserm n° 0000388FWA00005831) and the Commission Nationale Informatique et Libertés (CNIL n° 908,450 and n° 909,216). This study is registered in EudraCT (n°2013–000929-31). Written electronic informed consent to participate in the study was obtained from all subjects.

### Data collection

#### Food preparation behaviours

Based on published literature [5, 9, 10, 13, 15, 18, 19, 21, 23, 25, 38, 39], food preparation behaviours were captured by several dimensions: cooking frequency, daily time spent on food preparation, preparation from scratch, cooking skills, cooking enjoyment, willingness to cook better or more frequently, and kitchen equipment. Face validity was assessed by both experts and subjects. A team of multidisciplinary researchers (nutritionists, dieticians, economists and sociologists) developed the questionnaire. They evaluated whether the items were relevant to assess the measured concept, and only that concept, and whether they constituted a representative sample of a set of items describing the concept. Experts also evaluated the quality of the visible features of the items: length, items’ wording, and categories of response. Acceptability and feasibility were also measured in 100 subjects using specific questions on the perceived complexity and difficulty of filling in the questionnaire and whether the questionnaire was too long or any items were redundant, using a 4-point Likert scale from “I strongly disagree” to “I strongly agree”.

##### Frequency and time for meal preparation

Participants were asked who the main cook in the household was and how often they prepared meals during a typical week, including preparation of a cold dish or reheating a prepared dish (two or more times per day, once a day, several times a week but not every day, once a week, less than once a week, never). If participants answered never, no further questions regarding food preparation behaviours were asked. Then, participants were asked how much time (in minutes) they usually spent preparing meals, including cooking time. To assess average daily time preparing food, we multiplied the duration for meal preparation by the frequency and divided by 7.

##### Preparation from scratch

To assess preparation from scratch, i.e. use of raw or fresh ingredients that had no or minimal processing, participants were asked to assess their use of foods (fruit, vegetable, fish and meat) according to their processing level. Use of fruit or vegetables with no or minimal processing was assessed with the questions “Among the following fruits/vegetables, which ones do you use unpeeled, uncut, unprocessed?”. Ten groups of vegetables (1-tomato, pepper, eggplant; 2-cucumber, zucchini; 3-potato; 4-garlic, onion, shallot; 5-lettuce and other salad, endive, fresh spinach; 6-beets, carrots, celeriac, etc.; 7-beans, peas, etc.; 8-asparagus, celery, fennel, leeks, artichoke, etc.; 9-broccoli, cauliflower, cabbage, brussels sprouts, etc.; 10-mushrooms) and six groups of fruits (1-orange; 2-grapefruit, lemon; 3-apple, pear, quince, apricot, peach, plum, cherry; 4-grape, strawberry, raspberry, etc.; 5-banana, pineapple, kiwi, mango, etc.; 6-nut, hazelnut, chestnut, almond, etc) were proposed. Use of unprocessed fruit and use of unprocessed ‘tomato, pepper, eggplant’, ‘cucumber, zucchini’, ‘garlic, onions, shallots’ and potatoes were excluded from the score calculation as more than 95% of the participants used these items. When a participant self-reported the use of an unpeeled, uncut, unprocessed vegetable group, 1 point was allocated (Additional file 1: Table S1). For fish, participants had to determine what forms of fish, they usually use (whole fish not cleaned out, whole fish cleaned out, fish fillets, sliced, pavers or steaks, breaded fish). Participants also had to assess what forms of meat, they usually use (chunky uncut pieces, whole poultry not cleaned out, whole poultry cleaned out, cut poultry or meat, ready to cook poultry or meat such as ultra-processed meat and nuggets). Participants were allowed to select several types of fish and meat. When a participant self-reported the use of whole fish (cleaned out or not, regardless of the use or not of fish fillets, sliced, pavers or steaks), 2 points were allocated (Additional file 1: Table S1). When a participant self-reported the use of breaded fish and the use of other forms (whole fish, cleaned out or not, fish fillets, sliced, pavers or steaks), 1 point was allocated, whereas when participants only reported breaded fish or no other form of fish, no points were allocated. When a participant self-reported the use of chunky uncut pieces, whole poultry not cleaned out, whole poultry cleaned out, cut poultry or meat (even if participant also used ready to cook poultry or meat), 1 point was allocated whereas, when participants only reported ready to cook poultry or meat such as ultra-processed meat and nuggets, no points were allocated. A score of preparation from scratch was calculated from 0 to 12 points according to answers regarding use of raw vegetables, forms of fish and meat used (Additional file 1: Table S1).

##### Cooking skills

Participants were also asked to assess their cooking skills regarding 7 dishes (mashed potatoes; savoury pie or pizza; vegetable gratin; stewed meat or fish dish; omelette; vegetable soup; bread), 8 pastries and sweets (ice cream or sorbet; yogurt; pancakes or waffles; cakes or pastries; floating islands; chocolate mousse; macaroons; pie), 7 sauces (salad dressing; mayonnaise or aioli; garlic butter or herb butter; béchamel sauce; tomato sauce; hollandaise sauce; sauce by reduction) and 4 cooking techniques (scale and clear out a whole fish; fillet a whole fish; stuff meat or poultry; tie up a roast). Skills to cook dishes and pastries were evaluated using two types of questions, according to the dishes/pastries and sweets. For instance, to the question ‘Do you know how to make pancakes or waffles?’ participants could answer “Yes, I know”, “Yes, I know but only with ready for use pancake batter”, “No, I don’t know” and “I’ve never tried”. When a participant reported making the more complex variant of the dish/pastry/sweet, i.e. entirely homemade, without ready for use ingredients, 2 points were allocated whereas, when participants reported preparing the variant with ready for use ingredients, 1 point was allocated. When ready for use preparation does not exist for a dish/pastry, participants could answer “Yes, I know”, “No, I don’t know” and “I’ve never tried”. The same answers were proposed to evaluate cooking techniques. When a participant answered “Yes, I know”, 1 point was allocated. Skills regarding sauces were evaluated using the question “Among the following sauces, which ones do you know how to prepare?”. Points were allocated according to the skill complexity of the sauces and the number of sauces reported: 4 points for hollandaise sauce or sauce by reduction, 3 points for 3 or 4 simple sauces (mayonnaise, garlic butter, béchamel, tomato sauce), 2 points for 2 simple sauces, 1 point for 1 simple sauce, no point for salad dressing (Additional file 1: Table S1). A score of cooking skills was calculated from 0 to 41 points based on skills to make dishes, pastries, sauces and cooking techniques (Additional file 1: Table S1). Some dishes such as omelette and vegetable soup were not included in the score as more than 90% of the participants declared making these dishes.

##### Kitchen equipment

Kitchen equipment was assessed by the question “Is your kitchen equipped with the following utensils and appliances?”. Using data from French statistics on income and living conditions [40], seven utensils and appliances were proposed: pressure cooker, zester, baking pan, measuring cup, food processor, gas oven or electric oven. When a participant reported possessing kitchen equipment, 2 points were allocated, except for common equipment such as gas oven or electric furnace for which 1 point was allocated. Kitchen equipment was transformed into a score from 0 to 11 points (Additional file 1: Table S1).

##### Enjoyment for food preparation and willingness to cook better and more frequently

Participants were asked to assess their enjoyment of food preparation using the question: “Do you enjoy cooking?” (Yes, including daily meal preparation; Yes, but not daily meal preparation; No). They were also asked to assess their willingness to improve their cooking skills, whatever the skill level, and to cook more frequently using the following questions: “Do you wish to cook better?” (Yes/No), “Do you wish to cook more often?” (Yes/No).

#### Socio-economic position and demographic characteristics

All data used in this study were collected at 24 months after inclusion, which is close to the administration of the cooking questionnaire in month 22. Socio-economic position of participants was assessed using three indicators: education, income and occupation, using categories consistent with the French National Institute of Statistics definitions [41]. If subjects were unemployed or retired, we noted the occupational category of their last job. Occupation was recoded into 5 classes: manual and office worker, intermediate profession (technician, skilled employee, teacher, nurse, etc.), and managerial staff, self-employed (craftsman, shopkeeper, company manager, farmer) and never-employed (homemaker, student, disabled). Participants were asked their monthly household income, including salary, social benefits, family allowance and rental income. Household income per month was calculated by household units (HU). One HU was attributed for the first adult in the household, 0.5 for other persons aged 14 or older and 0.3 for children under 14 [42]. Categories used for monthly income were the following: <1200 €, 1200–1800 €, 1800–2700 € and >2700 € per HU, plus a category for individuals who were unwilling to answer. To assess educational level, participants gave their highest attained qualification. Educational level was recoded into four categories according to distribution throughout the entire sample: primary education, secondary education, undergraduate (corresponding to up to 3 years after the high school), and post-graduate (more than 3 years after the high school diploma). Demographic factors included gender, age, household composition (single, couple without a child, couple with ≥1 child, and household without a child but with ≥3 adults).

### Statistical analysis

Each score (preparation from scratch, cooking skills and kitchen equipment) was linearly transformed into values ranging from 0 to 10 to standardize ratings. Interactions between gender and the three socioeconomic indicators were tested, since literature has shown that gender is a much stronger determinant of food preparation behaviours than other socio-demographic variables [12, 16, 19–21]. All analyses were performed separately for men and women, since almost all gender interactions were significant (*P* interaction <0.01). Descriptive comparisons between sexes were performed using Student’s t test and the Chi-square test. Independent associations between SEP factors and food preparation behaviours were examined using multivariable logistic regression for categorical variables (frequency of meal preparation, enjoyment of cooking and willingness to cook better and more frequently) and analysis of covariance for daily time spent on meal preparation, preparation from scratch, cooking skills, kitchen equipment, with the most highly educated group, the highest income group and the managerial staff group as references. The three SEP indicators (education, income and occupation) were included simultaneously in the models. Models were also adjusted for age, household composition and whether or not the main cook in the household. First, we performed analyses on the whole sample by comparing SEP characteristics between regular cooks (individuals who cook at least once a day), occasional cooks (those who cook less than once a day but at least once a week) and non-cooks (those who cook less than once a week or never). Then, we studied relationships between socio-economic characteristics and food preparation behaviours among regular and occasional cooks. Sensitivity analyses were performed only in individuals who reported being the main cook in the household for all dimensions of food preparation behaviours. For these analyses, we used an identical approach as described above. Sensitivity analyses of the association of willingness to cook more frequently with SEP characteristics after adjustment for time spent for food preparation were also performed.

In addition, models using six categories of meal frequencies were produced. For men and women, weighting was calculated using the iterative proportional fitting procedure according to national census reports on age, birthplace, educational level, employment status, marital status, presence of children in the household and geographical area of residence [43]. Weighting was accounted for in all analyses. A *P*-value <0.05 was initially considered statistically significant. Then, to account for multiple comparisons, we calculated the Bonferroni correction, leading to a P-value <0.001. Data management and statistical analyses were performed using SAS (version 9.1; SAS Institute, Inc., Cary, NC, USA).

## Results

The vast majority of the sample reported that the questionnaire was not difficult (97%), annoying (93%) or too long (82%). Only a small proportion found it difficult (1%), annoying (6%) or too long (20%). A total of 64,466 individuals completed the questionnaire measuring cooking practices at 22 months, i.e. 66% of participants included in the Nutrinet-Santé cohort who were invited to respond. We excluded 1143 individuals who did not live in mainland France and 950 participants with missing sociodemographic and socioeconomic data, thus leaving 62,373 participants available for analysis (48,401 women and 13,972 men). Comparisons between participants in the analysis and total samples who were invited to fill in the questionnaire showed some differences. The percentages of younger people (6.0% in women and 2.5% in men vs. 10.2% and 6.3%), individuals living with at least one child (for women, 33.1% vs. 25.6%), never employed persons (5.1% in women and 2.1% in men vs. 6.1% and 3.2%) employees/manual workers (for women, 34.2% vs. 36.0%) and persons with income <1200 euros (16.5% in women and 10.9% in men vs. 18.4% and 12.5%) were lower in the final sample used for analyses than for the total sample (all *P* values <0.0001; data not shown). Regarding weighted data, percentages of participants with an undergraduate educational level, manual workers and office workers, never-employed and those in the lowest income class were higher among women than among men (Table 1).

**Table 1:** Demographic and socioeconomic characteristics of the sample (*n* = 48,401 women and *n* = 13,972 men)

	Raw data		*P*-value^#^	Weighted data^a^		*P*-value^#^
	Women	Men		Women	Men	
Education			*<0*.*0001*			*<0*.*0001*
Primary	2.9	3.9		30.2	24.1	
Secondary	33.1	34.9		44.2	50.9	
Under-graduate	32.4	23.5		13.3	10.7	
Post-graduate	31.6	37.7		12.3	14.3	
Occupation			*<0*.*0001*			*<0*.*0001*
Self-employed	2.7	4.8		5.2	9.9	
Never employed	5.1	2.1		5.1	3.6	
Manual worker, Office worker	34.2	16.8		54.1	39.2	
Intermediate profession	28.4	24.5		22.9	23.7	
Managerial staff	29.6	51.8		12.7	23.6	
Monthly household income per household unit			*<0*.*0001*			*<0*.*0001*
Unwilling to answer	12.0	6.2		15.1	9.9	
< 1200 euros	16.5	10.9		24.9	21.0	
1200–1800 euros	39.9	38.2		40.9	43.4	
1801–2700 euros	9.0	11.2		6.9	8.9	
> 2700 euros	22.6	33.5		12.2	16.8	
Age			*<0*.*0001*			*0.07*
18–24 years	6.0	2.5		10.6	10.8	
25–34 years	21.8	12.1		13.8	14.1	
35–54 years	40.9	33.9		35.9	39.1	
> 55 years	21.3	51.5		39.7	36.0	
Household composition			*<0*.*0001*			*0.22*
Single	17.8	14.6		16.6	14.6	
Couple without child	38.8	49.5		37.6	38.2	
Couple with ≥one child	33.1	26.3		36.6	36.9	
Household without child and with ≥3 adults	10.3	9.6		9.2	10.3	

^a^Weighting calculated according to national census reports on age, birthplace, educational level, employment status, marital status, presence of children in the household and geographical area of residence

^#^ *P*-value represented the overall significance of each variable (Type 3 analysis of effects)

Percentages of regular cooks (Women (W): 79.5% vs. Men (M): 43.6%; P < 0.0001), individuals who enjoy cooking including daily meal preparation (W:71.9% vs. M:69.9%; *P* = 0.04) were higher in women than in men while the percentages of non- and occasional cooks (W:17.9% vs. M:36.8%) and those who wished to cook more frequently (W:26.6% vs. M:33.7%; *P* < 0.0001) were lower (Table 2). Women spent more time for meal preparation and had higher scores of cooking skills preparation from scratch and kitchen equipment than men (Table 2).

**Table 2:** Food preparation behaviours in men and women^a^

	Women % or mean (SD)	Men % or mean (SD)	*P*-value^#^
All subjects			
Frequency of meal preparation	n = 48,401	n = 13,972	*<0*.*0001*
Non-cook (less than once/week or never)	2.6	19.6	
Occasional cook (less than once/day but at least once/week)	17.9	36.8	
Regular cook (once or more times/day)	79.5	43.6	
Occasional and regular cooks only	*n* = 47,556	*n* = 11,369	
Time for meal preparation (min/day)	42.0 (22.9)	27.9 (21.2)	*<0*.*0001*
Cooking skills (0–10 point score)	4.8 (1.2)	3.9 (1.6)	*<0*.*0001*
Preparation from scratch (0–10 point score)	5.5 (1.8)	5.1 (1.9)	*<0*.*0001*
Kitchen equipment (0–10 point score)	7.3 (1.9)	6.2 (2.4)	*<0*.*0001*
Enjoy cooking			*0.04*
Yes, including daily meal preparation	71.9	69.9	
Yes, but not daily meal preparation	18.3	18.0	
No	9.8	12.1	
Willingness to cook better			*0.61*
Yes	66.4	65.7	
No	33.6	34.3	
Willingness to cook more frequently			*<0*.*0001*
Yes	26.6	33.7	
No	73.4	66.3	

^a^time spent for food preparation, preparation from scratch cooking skills, kitchen equipment, enjoy cooking, willingness to cook better, willingness to cook more frequently were only assessed in occasional and regular cooks (*n* = 58,925)

^#^ *P*-value represented the overall significance of each variable (Type 3 analysis of effects)

Tables 3, 4, 5 presented independent associations of each socio-economic factor (education, income and occupation) with food preparation behaviours, after mutual adjustment for the others two SEP variables used and age, household composition and whether or not the participant is the main cook in the household.

**Table 3:** Associations between frequency of meal preparation and socioeconomic characteristics^a^

	Women *n* = 48,401					Men *n* = 13,972				
	Non-cooks vs. regular cooks		Occasional cooks vs. regular cooks		*P*-value^#^	Non-cooks vs. regular cooks		Occasional cooks vs. regular cooks		*P*-value^#^
	OR	CI 95%	OR	CI 95%		OR	CI 95%	OR	CI 95%	
Education					*0.001*					*0.0003*
Primary	*3.36*	*1.69;6.69*	0.90	0.69;1.17		*1.83*	*1.07;3.16*	1.21	0.79;1.84	
Secondary	*1.65*	*1.07;2.52*	1.06	0.93;1.20		*1.29*	*1.03;1.84*	0.91	0.73;1.13	
Under graduate	0.96	0.64;1.44	0.92	0.83;1.01		1.13	0.83;1.54	0.95	0.77;1.16	
Post graduate	1.00		1.00			1.00		1.00		
Occupation					*0.0005*					0.15
Self-employed	*0.08*	*0.02;0.30*	0.79	0.49;1.27		0.78	0.45;1.35	0.91	0.63;1.28	
Never employed	*0.30*	*0.11;0.77*	0.94	0.68;1.30		1.55	0.42;5.07	1.39	0.70;2.75	
Manual worker, office worker	*0.52*	*0.28;0.97*	0.88	0.74;1.05		0.80	0.56;1.13	0.88	0.67;1.15	
Intermediate profession	*0.45*	*0.21;0.98*	0.97	0.83;1.13		0.79	0.66;1.02	0.98	0.88;1.37	
Managerial staff	1.00		1.00			1.00		1.00		
Monthly household income per consumption unit					*0.0006*					0.01
Unwilling to answer	1.12	0.61;2.05	*0.87*	*0.55;0.99*		1.51	0.91;2.53	0.81	0.53;1.23	
< 1200 euros	2.01	0.98;3.65	*0.77*	*0.53;0.97*		1.32	0.84;2.07	0.90	0.65;1.27	
1200–1800 euros	1.14	0.68;1.90	0.87	0.75;1.02		1.43	1.09;1.88	0.94	0.75;1.03	
1801–2700 euros	0.90	0.49;1.64	0.88	0.71;1.09		0.72	0.56;1.09	0.78	0.56;1.09	
> 2700 euros	1.00		1.00			1.00		1.00		

^a^Multivariable logistic regression model in each sex, including the three socio-economic indicators (education, income and occupation) simultaneously, adjusted for age, household composition and whether or not the main cook in the household

#*P*-value represented the overall significance of each variable included in the model (Type 3 analysis of effects)

A *P*-value <0.001 was considered as statistically significant

**Table 4:** Associations between scores of daily time spent for meal preparation, preparation from scratch cooking skills and kitchen equipment, and socioeconomic characteristics^a^

	Women *n* = 47,556									Men n = 11,369								
	Daily time spent for meal preparation (min/day)			Preparation from scratch			Kitchen equipment			Daily time spent for meal preparation (min/day)			Preparation from scratch			Kitchen equipment		
	Mean	SE	*P*-value^#^	Mean	SE	*P*-value^#^	Mean	SE	*P*-value^#^	Mean	SE	*P*-value^#^	Mean	SE	*P*-value^#^	Mean	SE	*P*-value^#^
Education			*<0.0001*			0.009			0.01			*0.0006*			0.15			0.02
Primary	39.82	0.28		5.06	0.02		6.89	0.02		27.68	0.52		4.84	0.10		5.15	0.06	
Secondary	37.20	0.23		5.19	0.02		6.93	0.02		27.01	0.37		4.75	0.04		5.82	0.04	
Under graduate	35.36	0.33		5.23	0.02		7.01	0.03		25.53	0.63		4.79	0.05		5.92	0.07	
Post graduate	32.85	0.35		5.31	0.03		6.90	0.03		24.28	0.61		4.85	0.05		5.80	0.07	
Occupation			*<0.0001*			*<0.0001*			0.06			*<0.0001*			0.01			0.01
Self-employed	38.16	0.49		5.33	0.03		6.93	0.04		25.65	0.69		5.14	0.06		5.74	0.08	
Never employed	35.07	0.52		5.20	0.03		6.76	0.04		20.36	1.16		4.75	0.10		5.14	0.14	
Manual worker, Office worker	37.87	0.22		4.96	0.02		6.99	0.02		22.60	0.43		4.72	0.03		5.69	0.05	
Intermediate profession	35.65	0.25		5.17	0.02		7.01	0.02		24.84	0.48		4.73	0.04		5.90	0.05	
Managerial staff	34.53	0.34		5.21	0.02		6.96	0.02		27.34	0.52		4.92	0.05		5.91	0.06	
Monthly household income per consumption unit			*<0.0001*			0.008			*<0.0001*			*<0.0001*			0.002			0.09
Unwilling to answer	37.21	0.30		5.19	0.02		6.82	0.02		28.86	0.69		4.80	0.07		5.64	0.08	
< 1200 euros	37.11	0.27		5.15	0.02		6.77	0.02		25.98	0.51		4.56	0.05		5.88	0.06	
1200–1800 euros	35.67	0.22		5.28	0.02		6.95	0.02		24.59	0.41		4.88	0.04		5.81	0.04	
1801–2700 euros	36.24	0.43		5.32	0.03		7.13	0.03		23.59	0.72		4.83	0.06		5.41	0.08	
> 2700 euros	34.06	0.35		5.41	0.02		7.98	0.03		22.87	0.58		4.87	0.05		5.63	0.06	

^a^analysis of covariance in each sex, among regular and occasional cooks only, including the three socio-economic indicators (education, income and occupation) simultaneously, adjusted for age, household composition, and whether or not the main cook in the household

# *P*-value represented the overall significance of each variable included in the model (Type 3 analysis of effects)

A *P*-value <0.001 was considered as statistically significant

**Table 5:** Associations of willingness to cook more frequently with socio-economic characteristics^a^

	Women *n* = 47,556			Men *n* = 11,369		
	Willingness to cook more frequently (Yes vs. No)			Willingness to cook more frequently (Yes vs. No)		
	OR	CI 95%	*P*-value^#^	OR	CI 95%	*P*-value^#^
Educational level			*<0.0001*			*0.001*
Primary	*0.59*	*0.46;0.76*		*0.65*	*0.40;0.99*	
Secondary	*0.82*	*0.73;0.92*		*0.67*	*0.51;0.86*	
Under graduate	*0.90*	*0.84;0.98*		0.79	0.62;1.01	
Post graduate	1.00			1.00		
Occupational categories			*0.0001*			0.96
Self-employed	0.79	0.55;1.13		0.95	0.64;1;40	
Never employed	*0.89*	*0.66;0.97*		0.99	0.49;2.02	
Manual worker, Office worker	*0.96*	*0.85;0.99*		0.89	0.65;1.21	
Intermediate profession	1.01	0.91;1.12		0.99	0.79;1.23	
Managerial staff	1.00			1.00		
Monthly household income per consumption unit			*0.0006*			0.25
Unwilling to answer	0.98	0.80;1.22		1.05	0.69;1.51	
< 1200 euros	*0.85*	*0.72;0.99*		1.02	0.94;1.59	
1200–1800 euros	1.04	0.93;1.18		1.23	0.50;1.13	
1801–2700 euros	1.00	0.83;1.22		0.75	0.77;1.05	
> 2700 euros	1.00			1.00		

^a^Multivariable logistic regression model in each sex, among regular and occasional cooks only, including the three socio-economic indicators (education, income and occupation) simultaneously, adjusted for age, household composition, and whether or not the main cook in the household

# *P*-value represented the overall significance of each variable included in the model (Type 3 analysis of effects)

A *P*-value <0.001 was considered as statistically significant

In both sexes, individuals with primary and secondary education level were more likely to be non-cooks than those with the highest education level, in comparison to regular cooks (Table 3). In women, manual and office workers, self-employed and never-employed individuals were less likely to be non-cooks than managerial staff while individuals belonging to the two lowest income classes and those who were unwilling to provide their income level were less likely to be occasional cooks than those with the highest income, in comparison to regular cooks.

Among occasional and regular cooks, women and men with primary and secondary education level and those with the lowest income spent more daily time preparing food than those with the highest education level and those with the highest income (Table 4). In women, managerial staff spent less daily time preparing food than the other occupational categories, in particular manual and office workers and self-employed whereas, in men, managerial staff spent more daily time preparing food than the other occupation categories (Table 4). Female manual and office workers had lower score of preparation from scratch than female managerial staff and self-employed (Table 4). Women belonging to the lowest income class had a lower score of owning kitchen equipment than those with the highest income (Table 4).

Both women and men with primary and secondary education level, female manual and office workers, never-employed and women belonging to the lowest income class were less likely to wish to cook more frequently than those with the highest education level, managerial staff and those with the highest income, respectively (Table 5). No significant educational, occupational or income difference was found in cooking skills and willingness to cook better (Additional file 1: Table S2). Women belonging to the lowest income class were less likely to enjoy cooking daily meal preparation than those with the highest income while no difference was observed in men (Additional file 1: Table S2).

Sensitivity analysis that excluded individuals who were not the main cook in the household only modified the results regarding frequency of food preparation: significant associations with the three indicators became non-significant (data not shown). Regarding other indicators of food preparation behaviours, all associations remained significant but the strength of the associations was slightly lower than in analyses with the whole sample. Sensitivity analysis that examined meal frequency in 6 categories showed that female manual and office workers and women with low income were more likely to cook (two or more times per day or once a day), compared with those of the highest occupational categories, in univariate and multivariate analyses. Regarding education, the lowest educated individuals were more likely to self-declare extreme frequencies (never and two or more times per day), compared to higher educated persons. Analyses of the association of willingness to cook more frequently with socio-economic characteristics after adjustment for time spent for food preparation found that the association between willingness to cook more frequently and these indicators did not remain significant.

## Discussion

The present study addressed differences in food preparation behaviours according to socioeconomic groups in French adults using education, income and occupation as indicators of SEP. Compared with persons of high SEP, individuals at lower SEP (particularly women) spent more time preparing meals each week and consequently they did not wish to cook more often. Our findings therefore suggest that interventions promoting greater investment in meal preparation from low SEP women, without taking time scarcity and constraint into account, may not be effective. Considering the higher proportion of women than men responsible for food preparation in France, as in other European countries [16, 44], and consequently less confidence in cooking by men [21], interventions to improve low SEP men’s willingness to cook, and to improve their cooking confidence and cooking skills may be also useful. Indeed, in the UK, men on Jamie Oliver’s Ministry of Food course reported a significantly greater increase in cooking confidence than women after the course [45]. For other dimensions, each SEP indicator was associated with specific differences in food preparation behaviours suggesting that they underpin different facets of SEP [30, 33]. Owning kitchen equipment was only related to household income, while use of raw or fresh ingredients to prepare meals varied with occupation. This last finding suggests that it might be useful to implement nutritional interventions in female manual workers and office workers. For instance, interventions that uses their social networks to enhance the socialization around healthy eating and to provide advice, such as how to use fresh foods without investing too much time in preparation, may improve dietary intake of this population group.

In our study, no significant association between SEP and cooking skills was found, whatever the SEP indicator used in univariate and multivariate analyses, while results of previous work on this relationship are equivocal [6, 7, 21, 23, 46]. Due to strong cultural inheritance of French cuisine, cooking skills in France may be equally rooted in all socioeconomic classes [47, 48], unlike countries with less strong food culture. In addition, the increasing popularity of culinary TV shows and websites have developed leisure cooking activities [49] and consequently may lead to improved cooking skills and techniques in the general population, independently of SEP.

In line with Virudachalam et al. who found that less educated households were more likely to either always or never cook dinner at home, independently of sex [28], our study highlighted that lower educated individuals reported more extreme frequencies of food preparation (never and two or more times per day), compared to higher educated persons (results not shown). Findings regarding daily time spent on food preparation in previous studies are opposite [11, 16, 20]. Our results confirmed those of the French Time survey showing that low educated women spent more time on food preparation in a week [44]. Higher education, interpreted as greater knowledge, skills, and understanding of the conceptual and normative codes of a specific culture, may suggest the receipt of greater demands from others, resulting in preparing convenient and time efficient meals for oneself and others [34]. Although individuals with high education were more inclined to have healthy dietary intake [50], our findings seem to suggest that overall time spent cooking by the French population may not necessarily translate to healthy cooking and healthy dietary intake when cooking is a regular activity [51]. Compared to lower educated, high educated individuals may have sufficient nutrition knowledge and the capacity to develop other dietary behaviours, such as healthier food purchasing and food choices, but also better resources that compensate for the short time spent preparing healthy meals [29, 52].

Like education, possession of social capital (indicated by occupation) was associated with food preparation behaviours through lower frequency and time spent preparing meals. In line with previous work [11, 16, 19, 20, 28], including a French Time survey [44], female manual workers and office workers cooked more frequently and spent more time preparing meals in a week than managerial staff. This finding may be explained by lower daily working hours (in manual workers and office workers) or more flexible working hours (in self-employed) that allow them to free more time for domestic tasks such as meal preparation [53].

Less time preparing meals in female managerial staff may also reflect sharing of cooking responsibilities within the household over a week, rather than less time spent on each meal they cook, compared with manual workers and office workers. Indeed, unlike women, male manual workers and office workers spent less time for food preparation in a week than managerial staff, as in previous studies [16, 19]. This suggests that men in low occupational groups are more rooted in the traditional allocation of household tasks, with an unequal allocation between genders, than those in the high occupational category. In addition, female manual workers and office workers reported not wanting to cook more frequently, which confirms findings of a previous French study reporting that food preparation is perceived as a duty in low occupational categories [47], as they already cook frequently and do not see the need to do more. Indeed, after adjustment for time spent preparing food, the association between willingness to cook more frequently and occupation did not remain significant.

Our finding that female manual workers and office workers used slightly less raw or fresh ingredients to prepare meals than managerial staff is similar to studies on occupational disparities in dietary intake, in particularly a lower intake of vegetables and higher intake of processed foods has been reported in low occupational categories [50, 54]. Women with low occupational status might cook meals with fewer fresh vegetables and raw fish and meat, which indicate foods more likely to be prepared from scratch, compared with managerial staff, who tend to cook dishes based on other food groups. Another explanation could be that managerial staff associated more of the foods requiring cooked from scratch with notions of healthy eating efficacy and health conscious eating than manual and office workers [34].

As expected, income was the sole SEP indictor associated with owning kitchen equipment. Income has a direct impact on equipment through financial resources [33]. In line with previous studies [3, 19, 28, 31, 44], we found that individuals living in low income households cooked more frequently and spent more time preparing meals in a week compared with highest income households. Household income influenced the time spent preparing meals by substituting time with money, i.e. low income individuals are less able to substitute money for time and consequently to substitute higher quality prepared foods bought away from home, for home-cooked foods [19]. Low income women may not have life circumstances or material resources for adequate meal preparation, such as kitchen equipment, that would foster adaptation to high time demands [55]. This might contribute towards dietary inequalities by influencing the nutritional quality of prepared meals. We also found that women with low income were less likely to enjoy daily meal preparation and they did not have any desire to cook more often than women on a high income, as they already spent more time preparing meals. In a previous French study, meal preparation was perceived as a constraint for low income women compared to those with high income [47]. Adams et al. [16] underlined the differential effect of time pressures between working men and women on time spent for food preparation. This may explain why income disparities in enjoyment for meal preparation were only observed in women in our study.

Interpretation of the study’s findings should account for several limitations. Since the NutriNet-Santé Study is a voluntary cohort, sampling is not random and more subjects were women, belonged to high education and occupation groups and thus probably more concerned about healthy lifestyle and food preparation than the general population [56, 57]. However, previous analysis has shown geographic and socio-demographic diversity in participants at baseline, resembling the age and socioeconomic distribution of the French general population due to weighting [56]. Nevertheless, caution is needed when interpreting and generalizing findings. Differences in food preparation behaviours between SEP categories are probably greater in the general population. In addition, the web-based design could mitigate recruitment biases [58]. Indeed, a previous analysis of participants in this cohort showed that the exclusive use of the internet for data collection and follow-up may increase the proportion of population groups that are often underrepresented in volunteer cohorts such as men and older subjects [57]. In addition, the large sample size in the study may have been a constraint since significant results were found even when the difference between groups was small. However, the sample size and the diversity of collected data about demographic and socioeconomic factors enabled a highly accurate estimate and an adjustment for several confounders.

One strength of our study is the assessment of food preparation behaviours using an original questionnaire measuring various dimensions, while most previous studies have evaluated only one dimension. Food preparation behaviours are complex to define and measure [5, 15], therefore this questionnaire was carefully developed by experts, following the definition of one of the components of food literacy which fell into the “preparation” domain and that includes being able to prepare commonly available foods, efficiently use common pieces of kitchen equipment and having a sufficient repertoire of skills to adapt recipes to experiment with food and ingredients [39]. An extensive literature review on different indicators such as frequency and time spent preparing food, self-estimated cooking skills and knowledge, enjoyment of cooking, preparation from scratch or complex food preparation techniques was performed to develop the questionnaire. However, dimensions of food preparation used in our analysis were constructed using a posteriori approach (score computation) from descriptive data instead of a priori approach testing reliability of the questions and consequently the tool may be not adapted to other population. In addition, test–retest reliability was not assessed, which does not allow an estimation of the repeatability of the questionnaire.

Another limitation was that the data were self-reported, which may induce misreporting. Bias associated with social desirability is lower in studies using self-reported questionnaires, rather than face to face interviews, because it introduces distance between the investigator and the subject [59, 60]. Another limitation was that the “occupation” criterion cannot be reliably used for social groups outside of the paid workforce [33], including homemakers, disabled persons and students. Also, self-employed persons are difficult to classify, since this category is extremely heterogeneous and includes company managers, freelancers, shopkeepers, crafts persons and workers in informal sectors of the economy. As a result, comparison between their behaviours and those of the other categories may be biased, since such groups are extremely heterogeneous in terms of social status and relationships. However, comparison between the other occupational classes was interpreted in our study as a socio-economic gradient, from manual and office workers to managerial staff, as defined by the French National Institute of Statistics [41]. Also, personal income is a sensitive question and participants can be reluctant to provide such information, although this may have been overstated [33]. Since this SEP indicator is subject to greater non-response than other SEP questions, socio-economic differences could be incorrectly estimated.

## Conclusions

Despite an unfavourable context in France from reduced time spent preparing meals over recent decades, our findings do not show large socioeconomic disparities in food preparation behaviours. Unlike existing social inequalities in foods and nutrient intake in the French population [61, 62] consistent evidence of depreciation of food preparation behaviours in low SEP groups was not observed. Further research assessing the mediating effect of food preparation behaviours on the relationship between SEP and diet quality would be useful gain insight into the mechanisms of socioeconomic inequalities in diet. Our study reveals that low socio-economic populations, particularly for women, spend more time preparing food, but have no desire to cook more often, whatever the SEP indicators. In addition, simultaneous use of three socio-economic indicators highlighted distinct facets of social capital that may influence food preparation behaviours, such as less use of raw or fresh ingredients to prepare meals in female manual workers and office workers and better kitchen equipment in individuals with high income.

## Additional file

Additional file 1: Table S1. Calculation of scores of food preparation. Calculation of scores of food preparation from scratch, cooking skills and kitchen equipment. **Table S2.** Associations between scores of cooking skills, willingness to cook better and enjoy cooking, and socioeconomic characteristics. Analysis of covariance and multivariable logistic regression models in each sex, to assess associations between scores of cooking skills, willingness to cook better and enjoy cooking, and socioeconomic characteristics, between including the three socio-economic indicators (education, income and occupation) simultaneously, adjusted for age, household composition, and being or not the main cook in the household. (DOCX 50 kb)

## Abbreviations

HU: household units
SEP: socioeconomic position
