# Supplementary material for: Social disparities in food preparation behaviours: a DEDIPAC study
Source: Nutr J. 2017 Sep 20;16:62. doi: 10.1186/s12937-017-0281-2 (PMC5607511; doi:10.1186/s12937-017-0281-2)
Supplement: Additional file 1: Table S1. — Calculation of scores of food preparation. Calculation of scores of food preparation from scratch, cooking skills and kitchen equipment. Table S2. Associations between scores of cooking skills, willingness to cook better and enjoy cooking, and socioeconomic characteristics. Analysis of covariance and multivariable logistic regression models in each sex, to assess associations between scores of cooking skills, willingness to cook better and enjoy cooking, and socioeconomic characteristics, between including the three socio-economic indicators (education, income and occupation) simultaneously, adjusted for age, household composition, and being or not the main cook in the household. (DOCX 50 kb) [file 12937_2017_281_MOESM1_ESM.docx]

Table S1. Computation of scores of food preparation behaviors

| **Use of foods with no or minimal processing (from 0 to 12 points)** | Points |
| --- | --- |
| *Unpeeled, uncut, unprocessed vegetables* |  |
| Lettuce and other salad, endive, fresh spinach | 1 |
| Beets, carrots, celeriac, etc | 1 |
| Beans, peas, etc | 1 |
| Asparagus, celery, fennel, leeks, artichoke, etc | 1 |
| Broccoli, cauliflower, cabbage, Brussels sprouts, etc | 1 |
| Mushrooms | 1 |
| *Forms of fish used* |  |
| Use of whole fish not cleaned out (even if participant also used whole fish cleaned out or fish fillets, sliced, pavers or steaks) | 2 |
| Use of whole fish cleaned out (even if participant also used fish fillets, sliced, pavers or steaks) | 2 |
| Breaded fish (even if participant also used whole fish not cleaned out, whole fish cleaned out or fish fillets, sliced, pavers or steaks) | 1 |
| Breaded fish only or none | 0 |
| *Forms of meat used* |  |
| Chunky uncut pieces, whole poultry not cleaned out, whole poultry cleaned out, cut poultry or meat (even if participant also used ready to cook poultry or meat) | 1 |
| Ready to cook poultry or meat only (ultra-processed met and nuggets) or none | 0 |
| **Cooking skills (from 0 to 41 points)** |  |
| *Dishes* |  |
| Make bread | 2 |
| Make bread with a bread maker | 1 |
| Make mashed potatoes (with unprocessed potatoes) | 2 |
| Make instant mashed potatoes | 1 |
| Make savory pie or pizza with homemade pastry shell | 2 |
| Make savory pie or pizza with ready for use pastry shell | 1 |
| Make a vegetable gratin | 1 |
| Make a dish with stewed meat or fish | 1 |
| *Desserts and pastries* |  |
| Make ice cream or sorbet with or without ice cream maker | 1 |
| Make yogurt with or without yogurt-maker | 1 |
| Make pancakes or waffles with homemade pancake batter | 2 |
| Make pancakes or waffles with ready for use pancake batter | 1 |
| Make cakes or pastries with homemade pastry shell | 2 |
| Make cakes or pastries with ready for use pastry shell | 1 |
| Make floating islands with homemade custard | 2 |
| Make floating islands with ready for use custard | 1  1 |
| Make chocolate mousse | 1 |
| Make macaroons | 1 |
| Make pie with homemade pastry shell | 2 |
| Make pie with ready for use pastry shell | 1  1 |
| *Sauces* |  |
| Make hollandaise sauce or sauce by reduction (even if participant can make or not salad dressing) | 4 |
| Make 3 or 4 simple sauces* (even if participant can make or not salad dressing) | 3 |
| Make 2 simple sauces (even if participant can make or not salad dressing) | 2 |
| Make only 1 simple sauce (even if participant can make or not salad dressing) | 1 |
| Make salad dressing or none | 0 |
| *Cooking techniques* |  |
| Scale and clear out a whole fish | 1 |
| Fillet a whole fish | 1 |
| Stuff meat or poultry | 1 |
| Tie up a roast | 1 |
| **Kitchen equipment (from 0 to 11 points)** |  |
| Have pressure cooker | 2 |
| Have zester | 2 |
| Have baking pan | 2 |
| Have measuring cup | 2 |
| Have food processor | 2 |
| Have gas oven or electric furnace | 1 |

*Simple sauces: mayonnaise, garlic butter, bechamel, tomato sauce

Table S2. Associations between scores of cooking skills willingness to cook better and enjoy cooking, and socioeconomic characteristics

|  | Women n = 47,556 | | | | | | | | | | |  | Men n=11,369 | | | | | | | | | |  |
| --- | --- | --- | --- | --- | --- | --- | --- | --- | --- | --- | --- | --- | --- | --- | --- | --- | --- | --- | --- | --- | --- | --- | --- |
|  | Cooking skills* | | | | Willingness to cook better (Yes vs. No)** | | | Enjoy cooking including daily meal preparation vs. not enjoy** | | Enjoy cooking but not daily meal preparation vs. not enjoy | |  | Cooking skills* | | | Willingness to cook better** (Yes vs. No) | | | Enjoy cooking including daily meal preparation vs. not enjoy** | | Enjoy cooking but not daily meal preparation vs. not enjoy | |  |
|  | Mean | SE | P-value | OR | | CI 95 % | P-value | OR | CI 95 % | OR | CI 95 % | P-value | Mean | SE | P-value | OR | CI 95 % | P-value | OR | CI 95 % | OR | CI 95 % | P-value |
| **Education** |  |  | 0.004 |  | |  | 0.02 |  |  |  |  | 0.18 |  |  | 0.03 |  |  | 0.12 |  |  |  |  | 0.01 |
| Primary | 4.51 | 0.01 |  | 0.82 | | 0.68;1.07 |  | 0.95 | 0.65;1.37 | 1.07 | 0.80;1.45 |  | 3.47 | 0.04 |  | 0.60 | 0.38;0.92 |  | 0.35 | 0.18;0.71 | 0.78 | 0.42.1.38 |  |
| Secondary | 4.56 | 0.01 |  | 0.83 | | 0.74;0.93 |  | 0.85 | 0.70;1.04 | 0.91 | 0.77;1.08 |  | 3.82 | 0.02 |  | 0.88 | 0.72;1.09 |  | 0.74 | 0.51;1.08 | 1.02 | 0.76;1.14 |  |
| Under graduate | 4.59 | 0.01 |  | 0.93 | | 0.86;1.02 |  | 1.02 | 0.88;1.18 | 0.96 | 0.87;1.10 |  | 3.83 | 0.04 |  | 0.90 | 0.72;1.14 |  | 0.69 | 0.46;1.04 | 1.06 | 0.76;1.48 |  |
| Post graduate | 4.52 | 0.02 |  | 1.00 | |  |  | 1.00 |  | 1.00 |  |  | 3.73 | 0.04 |  | 1.00 |  |  | 1.00 |  | 1.00 |  |  |
|  |  |  |  |  | |  |  |  |  |  |  |  |  |  |  |  |  |  |  |  |  |  |  |
| **Occupation** |  |  | 0.005 |  | |  | 0.02 |  |  |  |  | 0.85 |  |  | 0.17 |  |  | 0.58 |  |  |  |  | 0.19 |
| Self-employed | 4.72 | 0.02 |  | 1.05 | | 0.78;1.42 |  | 1.45 | 0.78;2.68 | 1.27 | 0.74;2.12 |  | 4.02 | 0.05 |  | 0.85 | 0.57;1.28 |  | 1.04 | 0.55;1.94 | 0.81 | 0.46;1.41 |  |
| Never employed | 4.48 | 0.02 |  | 0.99 | | 0.64;1.53 |  | 0.95 | 0.54;1.67 | 1.05 | 0.64;1.70 |  | 3.79 | 0.09 |  | 0.59 | 0.23;1.53 |  | 0.19 | 0.06;0.65 | 0.48 | 0.16;1.48 |  |
| Manual worker, Office worker | 4.50 | 0.01 |  | 1.28 | | 1.09;1.49 |  | 0.88 | 0.67;1.15 | 0.97 | 0.78;1.21 |  | 3.65 | 0.03 |  | 0.84 | 0.63;1.12 |  | 0.77 | 0.47;1.25 | 0.85 | 0.56;1.29 |  |
| Intermediate profession | 4.51 | 0.01 |  | 1.08 | | 0.94;1.25 |  | 0.89 | 0.71;1.13 | 0.95 | 0.78;1.16 |  | 3.70 | 0.03 |  | 0.95 | 0.77;1.18 |  | 0.89 | 0.61;1.32 | 0.88 | 0.62;1.23 |  |
| Managerial staff | 4.52 | 0.01 |  | 1.00 | |  |  | 1.00 |  |  |  |  | 3.81 | 0.04 |  | 1.00 |  |  | 1.00 |  |  |  |  |
|  |  |  |  |  | |  |  |  |  |  |  |  |  |  |  |  |  |  |  |  |  |  |  |
| **Monthly household income per consumption unit** |  |  | 0.01 |  | |  | 0.43 |  |  |  |  | **0.0005** |  |  | 0.01 |  |  | 0.57 |  |  |  |  | 0.01 |
| Unwilling to answer | 4.56 | 0.02 |  | 1.02 | | 0.84;1.25 |  | 0.87 | 0.62;1.22 | 1.09 | 0.83;1.42 |  | 3.71 | 0.05 |  | 1.30 | 0.85;1.99 |  | 1.71 | 0.83;3.52 | 1.29 | 0.69;2.40 |  |
| < 1200 euros | 4.59 | 0.01 |  | 0.89 | | 0.75;1.07 |  | **0.63** | **0.45;0.86** | 0.91 | 0.72;1.15 |  | 3.86 | 0.03 |  | 1.06 | 0.72;1.57 |  | 3.08 | 1.58;5.99 | 1.67 | 0.88;3.15 |  |
| 1200-1800 euros | 4.56 | 0.01 |  | 1.03 | | 0.91;1.17 |  | **0.86** | **0.71;0.99** | 1.04 | 0.88;1.22 |  | 3.78 | 0.03 |  | 0.98 | 0.75;1.24 |  | 1.50 | 0.99;2.27 | 1.17 | 0.79;1.70 |  |
| 1801-2700 euros | 4.58 | 0.02 |  | 0.98 | | 0.81;1.17 |  | 0.70 | 0.52;0.95 | 0.87 | 0.62;1.43 |  | 3.60 | 0.05 |  | 0.86 | 0.59;1.26 |  | 0.98 | 0.53;1.81 | 0.80 | 0.45;1. |  |
| > 2700 euros | 4.53 | 0.02 |  | 1.00 | |  |  | 1.00 |  |  |  |  | 3.62 | 0.04 |  | 1.00 |  |  |  |  |  | 5.63 |  |

*analysis of covariance in each sex, including the three socio-economic indicators (education, income and occupation) simultaneously, adjusted for age, household composition, and being or not the main cook in the household

** Multivariable logistic regression model in each sex, including the three socio-economic indicators (education, income and occupation) simultaneously, adjusted for age, household composition, and being or not the main cook in the household
